# Intimate partner violence and associated factors among married adolescent girls and young women in the pastoralist community of South Ethiopia: is intimate partner violence associated with cultural phenomena?

**DOI:** 10.3389/fpubh.2024.1329699

**Published:** 2024-03-22

**Authors:** Ergudo Namer, Mulugeta Shegaze Shimbre, Amsalu Alagaw, Tamirat Gezahegn Guyo

**Affiliations:** ^1^School of Public Health, College of Medicine and Health Sciences, Arba Minch University, Arba Minch, Ethiopia; ^2^School of Public Health, Cheeloo College of Medicine, Shandong University, Jinan, Shandong, China; ^3^Department of Public Health, Arba Minch College of Health Sciences, Arba Minch, Ethiopia

**Keywords:** intimate partner violence, adolescent girls, young women, pastoralist community, South Ethiopia

## Abstract

**Background:**

Violence against women is a major public health problem that affects the physical, sexual, mental, and social wellbeing of more than one-third of all women worldwide. Hence the purpose of this study was to determine the prevalence of physical and sexual intimate partner violence (IPV) and associated factors among married adolescent girls and young women (AGYW) belonging to the pastoralist community of Dassenech district, South Omo Zone, South Ethiopia.

**Methods:**

A community-based cross-sectional survey was conducted among married AGYW in the Dassenech district from March 1, 2022, to April 1, 2022. A multi-stage sampling technique was adopted to select 545 participants. The data were collected using pre-tested and standardized WHO multi-country study tools. A binary logistic regression model was fitted to identify the independent predictors of physical and sexual intimate partner violence. The adjusted odds ratio (AOR) with a 95% confidence interval (CI) was used to measure the effect size, and finally, a p-value<0.05 was considered statistically significant.

**Results:**

The prevalence of physical IPV among AGYW belonging to the pastoralist community of Dassenech district was 44.1% (95% confidence interval (CI): 40%, 48%) and that of sexual IPV was 39.3% (95% CI: 35%, 43%). The husband only deciding for the household (AOR = 11.36; 95% CI: 6.97, 18.53), the father performing the Dimi cultural ceremony (AOR = 3.70; 95% CI: 2.22, 6.14), and frequent quarrels (AOR = 2.06; 95% CI: 1.07, 3.99) are significantly associated with physical IPV. Both partners drinking alcohol (AOR = 3.47; 95% CI: 1.94, 6.20), the husband only deciding for the household (AOR = 11.23; 95% CI: 6.91, 18.27), and frequent quarrels (AOR = 2.29; 95% CI: 1.15, 4.56) were factors significantly associated with sexual IPV.

**Conclusion:**

Physical and sexual intimate partner violence is a significant public health problem in the study area. Therefore, interventional measures to change the attitude of cultural leaders, providing education to married men and women on risky sexual behavior, and empowering women need to be prioritized to prevent the occurrence of this problem.

## Introduction

Intimate partner violence (IPV) is a major public health concern, a concealed social problem, and a human rights violation of women. This problem can negatively affect women's mental, physical, reproductive, and sexual health (1). The prevalence of IPV is assumed to be even higher among young women and adolescents (2). Marriage types like polygyny and arranged marriage can lead to different sexual, physical, and psychological harms for women (3). Polygyny is a type of polygamous marriage in which multiple women unite to a single man by marriage; in this case, women are oppressed and men are superior (4). Under international law, polygyny is a form of violence against women (5). In an arranged marriage, the family chooses who their daughter or son should marry (6). These types of marriages are very common among AGYW, as typically older men marry young women in Africa (7). The magnitude of IPV is significant among AGYW. Evidence from WHO's multicounty study shows that the overall magnitude of IPV among ever-partnered AGYW whose ages ranged from 15 to 24 years was 30% (8). Moreover, a study conducted using demographic and health survey (DHS) data from 30 developing countries determined that the prevalence of IPV was 28% among adolescents and 29% among young women (9). Evidence from a systematic review revealed that the prevalence of IPV in 11 Arab countries and showed that physical IPV ranged from 6% to 59% and sexual violence ranged from 3% to 40% (10). In sub-Saharan Africa (SSA), among AGYW, in the year preceding the study, the overall percentage of AGYW reporting IPV ranged from 6.5% in Comoros to 43.3% in Gabon (11). According to the Ethiopian Demographic Health Survey (EDHS) 2016, 64.2% of married AGYW aged 15–24 years experienced at least one form of violence, with 23.4% affected by physical violence and 8.8% subjected to sexual violence (12).

Intimate partner violence is a major public health problem that affects women's sexual, physical, mental, and social wellbeing, and young adults and adolescents are the major population group affected by such violence (13, 14). About 38% of all female homicides worldwide are carried out by intimate partners (15). It is estimated that each year, ~200,000 homicides occur annually in this age group globally, with most occurring in low- and middle-income countries (16). For young women in the age group of 10–29 years, violence is one of the main causes of death (17). In 2020, about 47,000 women and girls were killed globally by intimate partners or family members, with an average death occurring every 11 minutes (18). All aspects of life are impacted by IPV, including participation in social activities and even death (2, 19). IPV can even expose women to an increased risk of unplanned pregnancy (20). Adolescent girls and young women who have experienced partner violence are more likely to have abortions, depression, and contact HIV/AIDS and other sexually transmitted infections (21).

Understanding the determinants of IPV is essential for formulating effective strategies to prevent it. Previous studies showed that individual variables including age, educational status, childhood abuse or violence witnessed at home, and discriminatory beliefs about what men and women should do are associated with IPV (22, 23). Moreover, household decision-making dominated by men, poor socioeconomic status, heavy consumption of alcohol, unemployment, frequent quarrels, and unwanted first sexual intercourse are other identified factors associated with IPV (8, 24).

The Ethiopian national strategy for prevention and response to sexual and gender-based violence was developed in 2017 aiming at increasing community awareness through training, tea talks, home visits, and focused group discussion. But, there is no specific IPV guideline in the country (25). Keeping this in mind, previous studies (8) have recommended the need for additional studies on IPV (physical and sexual) among adolescents, girls, and young women, particularly in low- and middle-income countries such as Ethiopia, where the current study was done. In addition, the pastoralist community is the most marginalized in Ethiopia and consists of a population with different living standards than the non-pastoralist population. The magnitude and factors responsible for IPV identified among the non-pastoralist population cannot be applied to the pastoralist groups. There is a paucity of evidence on IPV in Ethiopia and none from the pastoralist communities such as those living in the current study area. Furthermore, the study explores cultural factors like the Dimi ceremony, which involves fathers preparing daughters for genital mutilation and marriage, which may affect the IPV status of adolescents and young women in the pastoralist community. In the Dassenech district, Dimi is the biggest cultural ceremony in the life of a man belonging to the Dassanech tribe. The purpose of the ceremony is to bless and celebrate his daughter's future marriage and fertility. When the man has gone through Dimi, he becomes an elder in the community. During the ceremony, about 30 smaller animals and 10 cattle will be slaughtered, and other stock will be traded for coffee. Women and men dress in animal fur capes to feast and dance, and the leaders of the village bless the girl (26). Furthermore, the experience of this cultural event exposes AGYW to early marriage, causes them to be pulled out of school, and decreases their work options, making them vulnerable to the risk of IPV. Therefore, this study aimed to assess the physical and sexual IPV and associated factors among AGYW in the pastoralist community of Dassenech district, South Ethiopia.

## Methods and materials

### Study design, study area, and period

**Figure 1:**
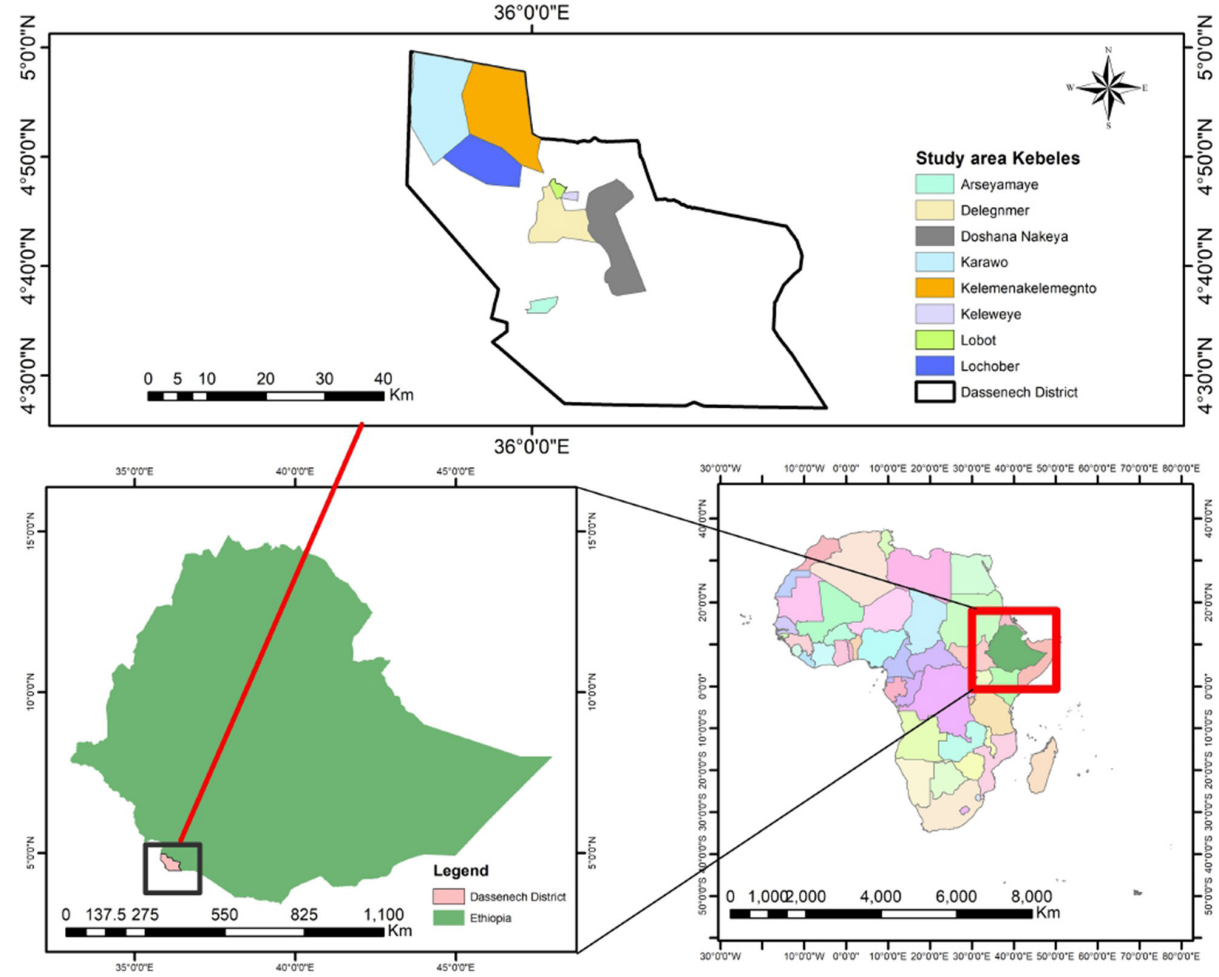
Study area map to assess intimate partner violence and associated factors among adolescent girls and young women in Dassenech district, South Ethiopia.

A community-based cross-sectional survey was conducted from March 1, 2022, to April 1, 2022, in the Dassenech district. This district is one of the pastoralist districts in the South Omo Zone in Ethiopia, located about 202 km away from Jinka town, an administrative center of the South Omo Zone, and 786 km from Addis Ababa, the capital city of Ethiopia. Dassenech district is bounded from the north by Gnagaton, the south and east by Kenya, and west by Hamer. There are 40 kebeles (small administrative units or neighborhoods) and Omorate is the administrative town of the Dassenech district (Figure 1). According to the 2022 report of the districts' municipality, the estimated total population of the district was 73,389 of which 37,429 were men, 35,960 were women, and 9,356 were AGYW whose age was < 25 years (27).

### Target population

The study target population included married AGYW of the Dassenech district, South Ethiopia, aged between 15 and 25 years. The following inclusion criteria were applied: (1) being a resident in a selected kebele of the Dassenech district for the last 6 months and (2) women who had ever been married or partnered, before the study period. AGYW who did not comply with the inclusion criteria were considered ineligible and excluded.

### Sample size determination and sampling procedure

The sample size was determined by Epi-Info version 7.2 by considering the following assumptions: 80% power, 95% CI, 1:1 ratio of exposed to unexposed, 42% (8) proportion of outcome (IPV) in unexposed (wanted sexual intercourse), and 58% proportion of outcome (IPV) in exposed (unwanted sexual intercourse) by taking women's first sexual intercourse as the exposure variable (8). The sample size was found to be 330 and was multiplied by 1.5 for design effect (330^*^1.5) because of the multi-stage nature of the sampling procedure. Then the calculated sample size was increased by 10% to account for the potential loss of the participants and sampling frame imperfection. Thus, the final sample size for the study was calculated to be 545.

**Figure 2:**
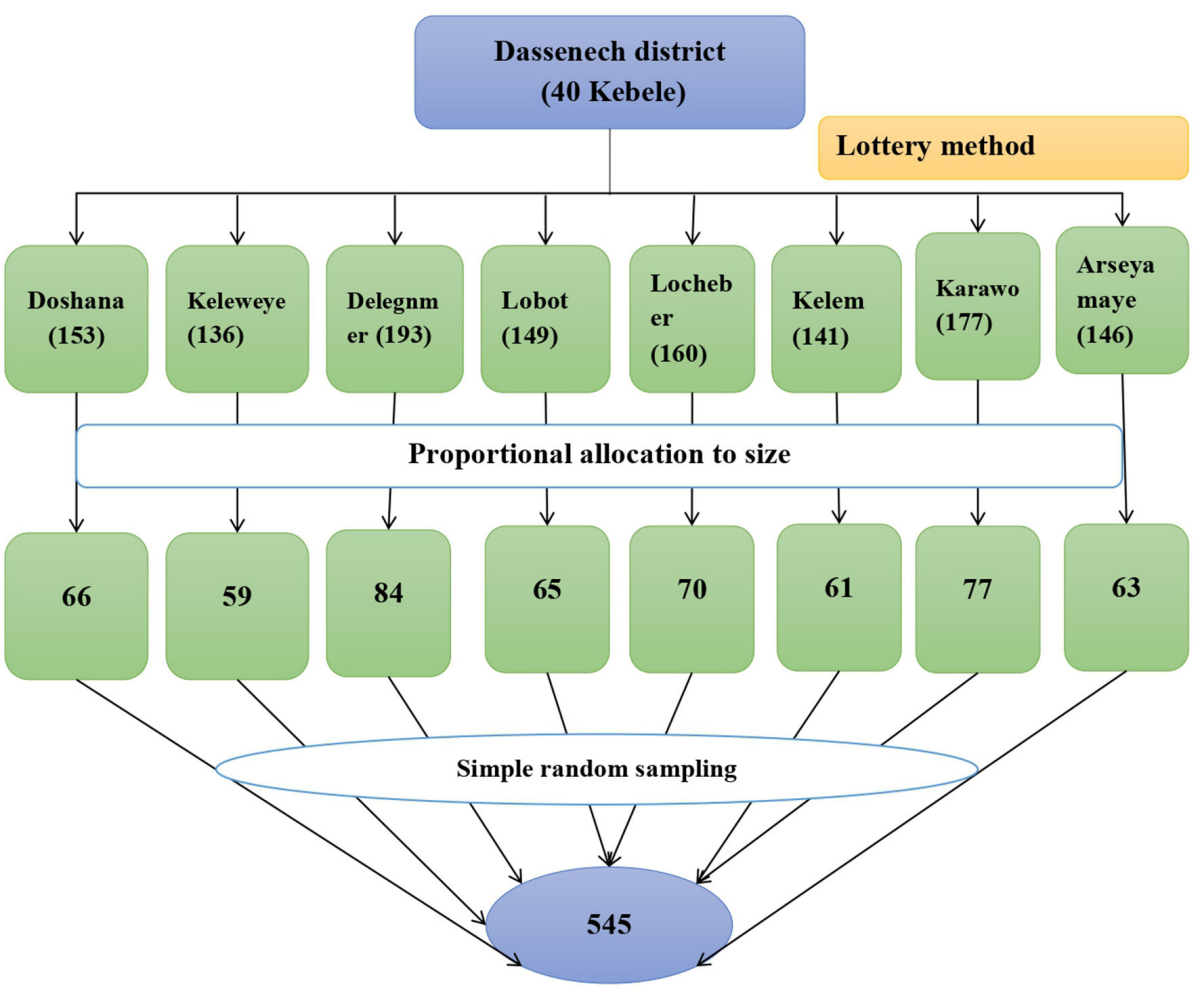
Schematic presentation of sampling procedure to intimate partner violence and associated factors among adolescent girls and young women in Dassenech district, South Ethiopia.

A multi-stage sampling procedure was used to select 545 respondents. In the Dassenech district, there are 40 kebeles of which eight kebeles (Arseyamaye, Delegnmer, Doshana, Karawo, Kelemenakelemegnto, Keleweye, Lobot, and Lochober) were selected by using a simple random sampling technique (lottery method). Then a sampling frame that contained all households with AGYW who were ever married or partnered before the study was prepared using a family-folder accessed from the health post of the selected kebeles. The calculated sample size was proportionally distributed to each of the included kebeles based on their population size. Finally, the study participants were selected using a simple random sampling technique (computer-generated random number) from households in the sampling frame. In a household where there are two eligible participants, one was randomly selected (Figure 2).

### Data collection tool, personnel, and procedure

Data were collected by standardized interviewer-administrated tools, which were adapted from the WHO Multicounty Study (28) and DHS 2016 (29). The questionnaire was first prepared in English, translated to Amharic, and then translated back to English to check for its validity and clarity. The questionnaire was pre-tested on 5% (*n* = 27) of the determined sample size and appropriate amendments were made to improve the consistency and clarity of the test instrument. Data were collected through face-to-face interviews by four female nurses holding a diploma and three public health professionals, all of whom completed a 1-day training program covering the objective of the study, survey operating procedures, consent forms, confidentiality, and data collection techniques. The data collection process was closely supervised by two field supervisors and the principal investigator, and the filled-out questionnaires were checked for completeness and consistency.

### Study variables

The dependent variables were physical intimate partner violence and sexual intimate partner violence. Independent variables were socio-demographic related (age, education status, marital status, occupation, household wealth index), before relationship related (childhood sexual abuse and women grew up with violence), relationship situation related (alcohol consumption, polygamy, partner fighting history, first sexual intercourse, number of parity, decision-making status), community and societal related (women's attitude toward IPV, Dimi cultural ceremony), and relationship quality related (controlling behavior, quarrel).

### Operational definition

Intimate partner physical violence was defined as the respondent answering “yes” to one or more deliberate acts of physical force or aggression, like pushing, slapping, throwing, punching, kicking, or killing young women and girls, or more acts like shouting, controlling, intimidating, or threatening the victim ever within the last 12 months before the survey (14).

Sexual intimate partner violence was considered to be committed when the study participant answered “yes” to any form of coercion, psychological intimidation, or force used to coerce an adolescent girl or young woman into performing a sexual act against her will, regardless of whether it was completed (13).

Partner fighting history refers to the partner's experience of fighting with others outside the household and can be categorized as “yes” if ever fighting or “no” if not fighting with anyone outside the household.

Childhood sexual abuse: All respondents were asked about the experience of sexual violence committed by anyone by asking the respondents if at any time in their life, as a child, they were forced in any way to have sexual intercourse or to perform any other sexual acts when they did not want to do so (29).

Decision-making status was ascertained through 10 questions describing the adolescent girls' and young women's involvement in making different household decisions. The response was provided a value of “1” if the respondent participated in the decision and “0” if she did not participate (30).

Household wealth index: Principal component analysis (PCA) was used to create the household wealth index, which was divided into quintiles ranging from 1 to 3 based on the quantity and type of goods owned by households as well as housing characteristics (drinking water, toilet facilities, flooring material, and availability of electricity) (31, 32).

### Statistical analysis

The data were entered into Epi-Data version 4.2 and then exported to the statistical package of social science (SPSS) version 26 for data management and analysis. Descriptive statistics, percentages, and frequencies for categorical variables, and mean with standard deviation for numerical variables were computed. The prevalence of IPV (physical or sexual) and respective 95% CI was calculated by dividing the number of cases (physical or sexual IPV) by total number of respondents using the STATA command (*prop IPV*). Bivariable logistic regression models were fitted to identify independent variables associated with each of the outcomes (intimate partner physical violence and intimate partner sexual violence). Variables with a *p* ≤ 0.25 were further included in the multivariable models. In multivariable logistic regression, variables with a *p* < 0.05 were identified as independent predictors of IPV, and the association between outcomes and independent variables was measured using adjusted odds ratios (AOR) with a 95% CI. Variance inflation factor (VIF) was used to assess multicollinearity, and Hosmer–Lemshow chi-square test was used to assess model goodness-of-fit. The significance level was set at 5%.

## Results

### Socio-demographic characteristics and house index of study participant

In the study, 545 married AGYW participated, with a 98% response rate. The mean age of the participants was 20.12 years with 2.21 SD years and the mean age of their husbands was 28 years with 4.68 SD years. The majority of the respondents (*n* = 456, 85.2%) did not attend formal education. While 40.8% (*n* = 218) were from households with poor wealth status, 35.9% (*n* = 192) were from households with a rich wealth status (Table 1).

**Table 1:** Socio-demographic, relationship-related, and community and social characteristics of married AGYW aged <25 years in the pastoralist community of Dassenech district, Southern Ethiopia, 2022 (*n* = 535).

**Variables**		**Frequency**	**Percent (%)**
Occupational status women	Housewife	373	69.7
	Other^*^	162	30.3
Educational status of women	No formal education	456	85.2
	Read and write	79	14.8
Educational status of husband	No formal education	456	85.2
	Read and write	79	14.8
Marital status	Married	467	87.3
	Divorced/ Widowed	68	12.7
Wealth index	Poor	218	40.7
	Middle	125	23.4
	Richer	192	35.9
Women grew up with violence	Yes	222	41.1
	No	313	58.5
Childhood sexual abuse	Yes	92	17.2
	No	443	82.8
Alcohol consumption	Neither drinks	212	39.6
	Only husband drinks	213	39.8
	Both drink	110	20.6
Parity	1–2 children	184	34.4
	3–4 children	288	53.8
	5 children and above	63	11.8
History of fighting	Yes	256	47.9
	No	279	52.1
Women's decision- making status	Husband	168	31.4
	Jointly	367	68.6
Controlling behavior	Yes	370	69.2
[-1pt]	No	165	30.8
Frequency of quarrel	Never	182	34.2
	Rarely	271	50.7
	Frequent	82	15.3
Women's attitudes toward IPV	Yes	230	43
	No	305	57
Dimi cultural ceremony	Yes	382	71.4
	No	153	28.6

AGYW, adolescent girl and young women; IPV, intimate partner violence. ^*^Daily labor, farmer.

### Characteristics of participants related to prior relationships, current relationships, quality of relationships, and community and social factors

It was revealed in the study that 222 (41.1%) married AGYW had grown up with violence while they were living in their family and 313 (59.9%) did not. Ninety-two (17.2%) married AGYW had experienced childhood sexual abuse. Of the participants, 213 (39.8%) husbands of married AGYW are drank alcohol, and 110 (20.6%) of both partners drank alcohol. Nearly one-third (34.6%) of the husbands are polygamous and have multiple sexual partners. According to the present study, more than half (317, 59.3%) of AGYW responded that their first sexual intercourse was wanted, 87 (16.3%) had unwanted sexual intercourse, and only 218 (40.75%) had their first sexual intercourse was forced by their partner. More than 50% of the participants (*n* = 288, 53.8%) had three to four children. Two hundred fifty-six (47.9%) husbands had a history of fighting outside the home and the remaining 279 (52.1%) did not have a history of fighting outside the home. Of the study participants, 367 (68.6%) husbands make decisions jointly with their partners, and in 165 (30.8%) households, the husbands make decisions on their own without allowing their partners to participate (Table 1).

The life behavior of more than two-thirds (*n* = 370, 69.2%) of AGYW had been controlled by their husbands and only for 165 (30.8%) women, their behavior was not controlled by their husbands. Of the AGYW who participated in the study, 271 (50.7%) had a rare frequency of quarrels in their relationship, and only 82 (15.3%) had frequent quarrels in their relationship. Of the participants, 230 (43%) AGYW accepted violence committed by their husbands as normal. Of the fathers of the participants, more than 70% (*n* = 382, 71.4%) had performed the Dimi cultural ceremony before marriage and the remaining one-fourth (*n* = 153, 28.6%) had not performed the Dimi cultural ceremony for their daughters (Table 1).

### Prevalence of intimate partner violence (physical and sexual)

The overall prevalence of intimate partner physical violence among married AGYW whose age was below 25 years in the past 12 months in the Dassenech district was 44.1% (95% CI: 40%, 48%) in which 38.1% (95% CI: 34.1 %, 42.3%) of the participants received slaps, 35.7% were kicked (95% CI: 31.7%, 39.9%), 21.1% were pushed (95% CI: 17.9%, 24.8%), 15.5% were hit with fist (95% CI: 12.7%, 18.8%), 10.3% were attacked with gun or knife (95% CI: 8.0%, 13.2%), and 1% were scalded or burned (95% CI: 0.4%, 2.2%). Among AGYW with no formal education, 46.3% (95% CI: 41.7%, 50.9%) had experienced physical IPV and among those with childhood sexual abuse history, more than half (55.4%; 95% CI: 45.1%, 65.3%) had experienced physical IPV. Moreover, among AGYW who had frequent quarrels, 51.2% (95% CI: 40.4%, 61.9%) experienced physical IPV.

The overall prevalence of sexual IPV among married AGYW whose age was below 25 years in the past 12 months was 39.3% (95% CI: 35%, 43%). Of this, 39.1% underwent sexual intercourse being afraid of it (95% CI: 35.0%, 43.3%), 12.3% had sexual intercourse when they did not want it (95% CI:9.8%, 15.4%), and 3.0% (95% CI: 1.8%, 4.8%) were degraded or humiliated during the sexual intercourse. Among AGYW with no formal education, 40.4% (95% CI: 35.9%, 44.9%) had experienced sexual IPV and among those with childhood sexual abuse history, 46.7% (95% CI: 36.7%, 57.0%) had experienced sexual IPV. Moreover, among AGYW who had frequent quarrels, 41.5% (95% CI: 31.3%, 52.5%) experienced sexual IPV.

Participants who experienced IPV were educated or counseled on reproductive health rights and human rights and how to fight for their rights. Moreover, they were encouraged to report the violence by exercising their right to government officials like those in the Women and Children Affairs department to get specific protection.

### Factors associated with intimate partner physical violence

In the bivariable logistics regression model, educational status of the women, occupational status of the women, polygamy, history of childhood sexual abuse, alcohol consumption status of the partners, decision-making status of the women, performing of Dimi cultural ceremony, and frequency of quarrels were selected for statistical modeling. It was observed that not attending any formal education (COR = 1.86; 95% CI: 1.12, 3.09; *p* = 0.017), being a housewife (COR = 1.77; 95% CI: 1.21, 2.59; *p* = 0.004), childhood sexual abuse (COR = 1.73; 95% CI: 1.10, 2.73; *p* = 0.02), alcohol consumption (COR = 2.41; 95% CI: 1.50, 3.86; *p* < 0.001), polygamy (COR = 1.85; 95% CI: 1.29, 2.65; *p* = 0.001), husband only makes the household decision (COR = 10.77; 95% CI: 6.91, 16.80; *p* < 0.001), Dimi cultural ceremony (COR = 4.33; 95% CI: 2.80, 6.72; *p* < 0.001), and frequency of quarrels (COR = 3.02; 95% CI: 1.75, 5.20; *p* < 0.001) were significantly associated with intimate partner physical violence (Table 2).

**Table 2:** Bivariable and multivariable analysis of factors associated with intimate partner physical violence among married adolescent girls aged <25 years in pastoralist community of Dassenech district, Southern Ethiopia 2022 (n = 535).

**Variables**		**Prevalence of physical IPV (95% CI)**	**COR (95% CI)**	***P*-value**	**AOR (95%CI)**	***P*-value**
Educational status of women	No formal education	46.3 (41.7, 50.9)	1.86 (1.12, 3.09)	0.017	1.49 (0.77, 2.89)	0.237
	Read and write	31.6 (22.2, 42.8)	1	1	1	
Occupational status of women	Housewife	48.3 (43.2, 53.3)	1.77 (1.21, 2.59)	0.004	1.16 (0.71, 1.89)	0.548
	Other^*^	34.6 (27.6, 42.2)	1	1	1	
Polygamy	Yes	54.1 (46.8, 61.1)	1.85 (1.29, 2.65)	0.001	1.18 (0.78, 1.85)	0.295
	No	38.9 (33.9, 44.1)	1	1	1	
Childhood sexual abuse	Yes	55.4 (45.1, 65.3)	1	1	1	
	No	41.8 (37.2, 46.4)	1.73 (1.10, 2.73)	0.017	1.47 (0.84, 2.56)	0.175
Alcohol consumption	Neither drink	34.9 (28.8, 41.6)	1	1	1	
	Husband drinks	46.9 (40.3, 53.7)	1.65 (1.12, 2.44)	0.012	1.06 (0.65, 1.73)	0.816
	Both drinks	56.4 (46.9, 65.4)	2.41 (1.50, 3.86)	< 0.001	1.28 (0.71, 2.31)	0.408
Decision-making status of women	Husband	80.4 (73.6, 85.7)	10.77 (6.91, 16.80)	< 0.001	11.36 (6.97, 18.53)	< 0.001
	Jointly	27.5 (23.2, 32.3)	1	1	1	
Dimi cultural ceremony	Yes	53.4 (48.4, 58.4)	4.33 (2.80, 6.72)	< 0.001	3.70 (2.22, 6.14)	< 0.001
	No	20.9 (15.2, 28.1)	1	1	1	
Frequency of quarrel	Never	25.8 (20.0, 32.7)	1	1	1	
	Rarely	54.2 (48.3, 60.1)	3.41 (2.26, 5.13)	< 0.001	4.12 (2.51, 6.77)	< 0.001
	Frequent	51.2 (40.4, 61.9)	3.02 (1.75, 5.20)	< 0.001	2.06 (1.07, 3.99)	0.031

^*^Other daily labor, farmer. AOR, adjusted odds ratio; CI, confidence interval; COR, crude odds ratio; IPV, intimate partner violence.

In multivariable analysis, the decision-making status of women, Dimi culture ceremony, and the frequency of quarrels were significantly associated with physical violence (*p* < 0.05). Participants whose husbands only made household decisions had more than 10 times (AOR = 11.36; 95% CI: 6.97, 18.53; *P* < 0.001) increased odds of being subjected to physical IPV than their counterparts. Participants who participated in the Dimi culture ceremony had 3.7 times (AOR = 3.70; 95% CI: 2.22, 6.14; *P* < 0.001) increased odds of experiencing physical violence than their counterparts. Participants with a rare frequency of quarrels (AOR = 4.12; 95% CI: 2.51, 6.77; *P* < 0.001) and frequent quarrels (AOR = 2.06; 95% CI: 1.07, 3.99; *P* = 0.031) had four and three times increased odds of receiving physical IPV than their counterparts, respectively (Table 2).

### Factors associated with intimate partner sexual violence

In the bivariable logistics regression model, educational status of the women, occupational status of the women, polygamy, history of childhood sexual abuse, alcohol consumption status of the partners, decision-making status of the women, performing of Dimi cultural ceremony, and frequency of quarrels were selected for the statistical model. It was observed that not attending education (COR = 1.38; 95% CI: 0.83, 2.29; *p* = 0.213), being a housewife (COR = 1.62; 95% CI: 1.09,2.39; *p* = 0.016), childhood sexual abuse (COR = 1.45; 95% CI: 0.92, 2.28; *p* = 0.107), alcohol consumption (COR = 3.98; 95% CI: 2.45, 6.48; *p* < 0.001), polygamy (COR = 1.58; 95% CI: 1.10, 2.28; *p* = 0.013), husband only made the household decision (COR = 8.80; 95% CI: 5.79, 13.36; *p* < 0.001), Dimi cultural ceremony (COR = 2.72; 95% CI: 1.78, 4.16; *P* < 0.001), and frequency of quarrel (COR = 3.08; 95% CI: 1.73, 5.49; *P* < 0.001) were associated with intimate partner sexual violence (*P* < 0.25) and were candidates for multivariable logistics regression analysis (Table 3).

**Table 3:** Bivariable and multivariable analyses of factors associated with intimate partner sexual violence among married adolescent girls aged <25 years in pastoralist community of Dassenech district, Southern, Ethiopia 2022 (*n* = 535).

**Variables**		**Prevalence of sexual IPV (95% CI)**	**COR (95% CI)**	***P*-value**	**AOR (95%CI)**	***P*-value**
Educational status of women	No formal education	40.4 (35.9, 44.9)	1.38 (0.83, 2.29)	0.213	0.94 (0.49, 1.83)	0.865
	Read and write	32.9 (23.4, 44.1)	1	1	1	
Occupational status of women	Housewife	42.6 (37.7, 47.7)	1.62 (1.09, 2.39)	0.016	1.15 (0.70, 1.88)	0.577
	Other^*^	31.5 (24.8, 39.1)	1	1	1	
Polygamy	Yes	46.5 (39.4, 53.7)	1.58 (1.10, 2.28)	0.013	0.99 (0.63, 1.57)	0.978
	No	35.4 (30.6, 40.6)	1	1	1	
Childhood sexual abuse	Yes	46.7 (36.7, 57.0)	1.45 (0.92, 2.28)	0.107	1.30 (0.74, 2.27)	0.363
	No	37.7 (33.3, 42.3)	1	1	1	
Alcohol consumption	Neither drink	27.4 (21.8, 33.8)	1	1	1	
	Husband drinks	40.4 (34.0, 47.1)	1.80 (1.20, 2.70)	0.005	1.34 (0.82, 2.18)	0.243
	Both drinks	60.0 (50.5, 68.8)	3.98 (2.45, 6.48)	< 0.001	3.47 (1.94, 6.20)	< 0.001
Decision-making status of women	Husband	73.2 (66.0, 79.4)	8.80 (5.79, 13.36)	< 0.001	11.23 (6.91, 18.27)	< 0.001
	Jointly	23.7 (19.6, 28.3)	1	1	1	
Dimi cultural ceremony	Yes	45.5 (40.6, 50.6)	2.72 (1.78, 4.16)	< 0.001	1.57 (0.94, 2.65)	0.087
	No	23.5 (17.4, 30.9)	1	1	1	
Frequency of quarrel	Never	18.7 (13.6, 25.0)	1	1	1	
	Rarely	52.4 (46.4, 58.3)	4.79 (3.08, 7.46)	< 0.001	6.20 (3.62, 10.58)	< 0.001
	Frequent	41.5 (31.3, 52.5)	3.08 (1.73, 5.49)	< 0.001	2.29 (1.15, 4.56)	0.018

^*^Other daily labor, farmer. AOR, adjusted odds ratio; CI, confidence interval; COR, crude odds ratio; IPV, intimate partner violence.

In multivariable analysis, alcohol consumption, decision-making status of women, and frequency of quarrel were factors significantly associated with sexual violence. Adolescent girls and young women who drank alcohol with their partners were 3.47 times (AOR = 3.47; 95% CI: 1.94, 6.20; *P* < 0.001) more likely to experience sexual violence than those who did not drink alcohol. Participants whose husbands only decide for the household had more than 10 times (AOR = 11.23; 95% CI: 6.91, 18.27; *P* < 0.001) increased odds of being subjected to sexual IPV than their counterparts. Participants with a rare frequency of quarrels (AOR = 6.20; 95% CI: 3.62, 10.58; *P* < 0.001) and frequent quarrels (AOR = 2.29; 95% CI: 1.15, 4.56; *P* = 0.018) had six and two times increased odds of experiencing sexual IPV than their counterparts, respectively (Table 3).

## Discussion

### Prevalence of IPV

In this study, the prevalence of physical IPV among married AGYW in the Dassenech district in the last 12 months was 44.1% while the prevalence of sexual IPV was 39.3%. The findings of the current study were in line with the study conducted in 27 sub-Saharan African countries, which reported prevalence of physical or sexual IPV as ranging from 6.5% in Comoros to 43.3% in Gabon and a median prevalence of combined IPV (physical and sexual) against AGYW of 25.2% (33). It is also consistent with the study performed in sub-Saharan Africa, in which IPV ranged from 7% to 67% (8), and the study done in Alexandria in which a prevalence of 50.3% of physical IPV and 37.1% of sexual IPV was reported (34). The prevalence of IPV in the current study was higher than the study conducted in Brazil, which reported physical IPV at 16.7% and sexual IPV at 29% (35). In Egypt, the reported prevalence of physical IPV was 15.2% and that of sexual IPV 17.8% (30). In Uganda, the reported physical IPV was 24.8% and sexual IPV was 29% (36). The study done in Ethiopia using EDHS 2016 noted the prevalence of physical IPV to be 23.4% and sexual IPV to be 8.8% (12). The discrepancy could be attributed to differences in the socio-demographic status of the community and socioeconomic and cultural differences in pastoralist communities as well as differences in data collection time. Another possible reason for the difference might be the difference in the study area or settings. For example, the studies done in Brazil (35), Alexandria (Egypt) (34), and Uganda (36) were conducted at the health facilities such as health centers and hospitals. In addition, studies conducted in sub-Saharan Africa (8, 33) and Ethiopia (12) were done at the country level, which included a very large sample size. Moreover, the discrepancy in the prevalence might be due to differences in the study population as previous studies conducted in Brazil (35) included adolescents and older women (age>20 years), that in Uganda (36) included teenagers, that in Alexandria (34) included only adolescents, and that in upper Egypt (30) included ever married women, while the current study included AGYW aged 15–25 years.

### Relationship situation-related factors

The evidence from the current study showed that alcohol consumption by both partners was significantly associated with sexual IPV. It revealed that AGYW who drank alcohol with their partners were three times more likely to experience sexual IPV than those who did not drink alcohol. This finding is consistent with studies conducted in nine African countries (8) and those in the United States (Baltimore, Maryland), India, China, Nigeria, and South Africa (37). This might be due to the association between alcohol consumption and intimate IPV, which could be mediated by anti-social behavior, suggesting that drinking may act as a trigger for physical IPV and that individuals with a tendency toward anti-social behavior may be more prone to engaging in violent acts while under the influence of alcohol. Additionally, alcohol impairs judgment and lowers inhibitions, potentially leading to increased aggression and violent behavior in intimate partner relationships (38). IPV is a significant factor in addressing the issue of alcohol consumption among young women and adolescents. Moreover, existing alcohol abuse programs should also increase awareness about its link to violence to mitigate its impact on intimate partner violence (39).

The current study reported that participants whose husbands only made decisions in the household had more than 20 times higher odds of engaging in IPV (physical and sexual) than their counterparts. This finding is in line with studies conducted in sub-Saharan Africa (40) and Bangladesh (41). The possible reason for this might be due to the low level of empowerment of women (12.1%) (40) in sub-Saharan Africa, and women who are less empowered cannot have reasonable and better communication with their partners. Moreover, less empowered women are unable to fight peacefully for their rights, such as for their involvement in household decision-making; rather, they oppose the decisions taken by their husbands, and the men respond with violence. This finding implies that the elimination of IPV among AGYW in the Dassenech district can be achieved through promoting and improving women's involvement in decision-making on major household issues.

### Community and society-related factors

The result of this study showed that fathers of AGYW performing Dimi cultural ceremonies were significantly prone to physical IPV. Adolescent girls and young women whose fathers performed a Dimi cultural ceremony were nearly 4-fold times more likely to experience physical violence as compared to their counterparts. The possible explanation might be the common practice of arranged marriage in different parts of Ethiopia, which limits education and development and further increases physical IPV (8). Furthermore, the Dassanech district emphasizes female genital mutilation (FGM) as a cultural identity marker, excluding non-cut women from the ‘Dimi' culture and preventing marriage between the Dassenechs (42). The Dimi ceremony is a significant man's life ritual, with the father celebrating and blessing the future marriage and fertility of his daughter after FGM and passing on the blessings to future daughters. Similarly, previous studies indicate that women undergoing FGM often experience greater dissatisfaction with their marital and sexual relationships compared to non-FGM women, and this might lead to frequent disagreement between the couples, which in turn exposes them to IPV (43). This finding stresses the need to strengthen the execution of programs and policies aimed at overcoming IPV through prevention and fighting against harmful traditional practices such as FGM, which would help the district contribute to improving global maternal health.

### Relationship quality-related factors

The present study showed that the frequency of quarrels was significantly associated with IPV (physical and sexual) among adolescents and young women in the study area. Participants who had a rare frequency of quarrels and frequent quarrels had four and three times higher odds of experiencing physical IPV than their counterparts, respectively. Similarly, participants who had a rare frequency of quarrels and frequent quarrels had six and two times higher odds of physical IPV than their counterparts, respectively. This finding is in line with studies conducted in nine African countries (8) and South Africa (44). It is also consistent with the study done in Nepal, (45) which stated that women involved in day-to-day quarrels had an increased odds of experiencing IPV. This study does not indicate whether marital conflict (quarrels) precedes or follows domestic violence; possibly, frequent quarrels can lead to decreased love and respect for partners, resulting in increased confrontation. The study underscores the significance of initiating early prevention measures to minimize the occurrence of IPV through the reduction of quarrels and the promotion of solving problems through discussions.

### Public health and policy implication

The findings from the current study are crucial for policy and practice in the Dassenech district, aligning with the SDG's 2030 target of eliminating IPV. The Ethiopian government and non-governmental organizations should prioritize the prevention of IPV, particularly in high-prevalence areas such as the pastoralist community of Dassenech district, by providing legal support for women's education and economic assistance and including health information provision. Additionally, strict enforcement of laws prohibiting IPV in the district is highly needed. Finally, a community mobilization targeting community key informants by the government in the district as well as the zone is also crucial.

### Strengths and limitations

The statistical analyses conducted on a large sample in the current study support the precision of the findings. There was the likelihood of recall bias from the respondents regarding their experience of violence and recalling the violence-related events that happened in the community. The study can also be affected by social desirability bias due to social and cultural norms that promote the acceptance of violence. In the present study, the frequency of IPV was not assessed and a single event as well as a frequent events of IPV were given equal weight. In addition, as the study used a cross-sectional methodology, the outcome might be frequent and the AOR could overestimate the magnitude of the association.

## Conclusion

Intimate partner violence is highly prevalent among married AGYW in the pastoralist community of the Dassenech district. A monopolized household decision-making by the husband, performing the Dimi cultural ceremony, and frequent quarrels were significant factors that were positively associated with physical IPV. Alcohol consumption by both partners, monopolized household decision-making by the husband, and frequent quarrels were significantly associated with sexual IPV. Therefore, special attention and urgent interventions are needed to reduce physical and sexual IPV among married AGYW. Interventional measures such as information, education, and communication (IEC) to change the attitude of the abuser, empower women, enhance school participation, and train key informants and cultural leaders on the adverse effect of IPV on AGYW are necessary. Future research that identifies the frequency of violence and the effect of other important factors is highly recommended.

## Data availability statement

The original contributions presented in the study are included in the article/supplementary material, further inquiries can be directed to the corresponding author.

## Ethics statement

All the study procedures were done according to the ethical guidelines outlined in the Helsinki Declaration. Before the start of data collection, ethical approval was obtained from the Arba Minch University Institutional Research Ethics Review Board (IRB) (reference number: IRB/37/2022). The School of Public Health wrote a support letter to the South Omo Zone Health Department, Dassenech District Health Office, and Kebele administration. A brief explanation was given about the aim of the study, the study procedure, benefits and possible risks, and the participant's rights to all the study participants. Then informed consent was obtained from each study participant before asking for any information from them. For minors (age < 18 years), a written informed consent has been obtained from parents/legal guardians and assent was obtained from participants. None of the study participants was obligated to participate in the study without his or her consent. They were informed that they have a full right to discontinue the study at any time if they feel uncomfortable with it. All the collected data were kept confidential, and the names or any personal identifiers of the study participants were not included in the data collection.

## Author contributions

EN: Writing—review & editing, Writing—original draft, Visualization, Validation, Software, Resources, Project administration, Methodology, Investigation, Funding acquisition, Formal analysis, Data curation, Conceptualization. MS: Writing—review & editing, Visualization, Validation, Supervision, Methodology, Formal analysis, Data curation, Conceptualization. AA: Writing—review & editing, Visualization, Validation, Supervision, Methodology, Formal analysis, Conceptualization. TG: Writing—original draft, Software, Conceptualization, Writing—review & editing, Visualization, Validation, Methodology, Formal analysis.
